# Housecleaning of pyrimidine nucleotide pool coordinates metabolic adaptation of nongrowing *Mycobacterium tuberculosis*

**DOI:** 10.1080/22221751.2018.1559706

**Published:** 2019-01-16

**Authors:** Kun-Xiong Shi, Yong-Kai Wu, Bi-Kui Tang, Guo-Ping Zhao, Liang-Dong Lyu

**Affiliations:** a Key Laboratory of Medical Molecular Virology of MOE/MOH, School of Basic Medical Sciences, Fudan University, Shanghai, People’s Republic of China; b Department of Microbiology, School of Life Sciences, Fudan University, Shanghai, People’s Republic of China; c Department of Life Science, Bengbu Medical College, Bengbu, People’s Republic of China; d Department of Microbiology and Li Ka Shing Institute of Health Sciences, The Chinese University of Hong Kong, Prince of Wales Hospital, New Territories, Hong Kong, People’s Republic of China; e CAS-Key Laboratory of Synthetic Biology, Shanghai Institute of Plant Physiology and Ecology, Chinese Academy of Sciences, Shanghai, People’s Republic of China

**Keywords:** *Mycobacterium tuberculosis*, dormancy, nongrowing state, pyrimidine metabolism, mazG

## Abstract

The ability of *Mycobacterium tuberculosis* (*Mtb*) to adopt a slowly growing or nongrowing state within the host plays a critical role for the bacilli to persist in the face of a prolonged multidrug therapy, establish latency and sustain chronic infection. In our previous study, we revealed that genome maintenance via MazG-mediated elimination of oxidized dCTP contributes to the antibiotic tolerance of nongrowing *Mtb.* Here, we provide evidence that housecleaning of pyrimidine nucleotide pool via MazG coordinates metabolic adaptation of *Mtb* to nongrowing state. We found that the *ΔmazG* mutant fails to maintain a nongrowing and metabolic quiescence state under dormancy models *in vitro*. To investigate bacterial metabolic changes during infection, we employed RNA-seq to compare the global transcriptional response of wild-type *Mtb* and the *ΔmazG* mutant after infection of macrophages. Pathway enrichment analyses of the differentially regulated genes indicate that the deletion of *mazG* in *Mtb* not only results in DNA instability, but also perturbs pyrimidine metabolism, iron and carbon source uptake, catabolism of propionate and TCA cycle. Moreover, these transcriptional signatures reflect anticipatory metabolism and regulatory activities observed during cell cycle re-entry in the *ΔmazG* mutant. Taken together, these results provide evidence that pyrimidine metabolism is a metabolic checkpoint during mycobacterial adaptation to nongrowing state.

Despite the availability of chemotherapy, tuberculosis remains a leading cause of death due to a single bacterium infection. Increasing evidence shows that the ability of *Mycobacterium tuberculosis* (*Mtb,* the causative agent of tuberculosis) to adopt a nongrowing state within the host plays a critical role for the bacilli to persist in the face of a prolonged multidrug therapy, establish latency and sustain chronic infection. Therefore, understanding the molecular events underlying growth control and metabolic adaptation of nongrowing *Mtb* is believed to be particularly important for the development of new therapeutic strategies [1,2]. In our previous studies, we showed that elimination of oxidized dCTP via NTP pyrophosphohydrolase MazG is required for the persistence of *Mtb* during chronic infection of mouse and contributed to antibiotic tolerance of stationary-phase culture and intracellular *Mtb* [3,4]. These results established that housecleaning of the pyrimidine nucleotide pool plays a crucial role in *Mtb* genome maintenance under stress environments. Given that all of these phenotypes are akin to a nongrowing state, we speculate that *mazG* may implicate in mycobacterial adaptation to growth-limiting environments. In this preliminary data report, we provide evidence that housecleaning of pyrimidine nucleotide pool coordinates metabolic adaptation of nongrowing *Mtb*.

To test whether *mazG* is required for mycobacterial adaptation to nongrowing state, we measured the survival of wild-type *Mtb* and the Δ*mazG* mutant under hypoxic and nutrient-starvation conditions, i.e. the two *in vitro* models of mycobacterial dormancy. Under both conditions, while the wild-type *Mtb* and the complemented Δ*mazG* mutant maintained slightly decreased level (Figure 1(a)) or the same level (Figure 1(b)) of colony forming units (CFUs) during the course of treatment, the Δ*mazG* mutants exhibited a survival advantage over that of the wild type (Figure 1(a,b)). Most dramatically, the deletion of *mazG* results in a CFU counts 10 times higher than that of wild-type *Mtb* after 5 weeks hypoxic treatment (Figure 1(a)), consistent with the results of a previous transposon library-screening study showing that inactivation of *Mtb mazG* (Rv1021, which was annotated as a conserved hypothetical protein in the Supporting Information of Ref. [5]) results in a survival advantage phenotype under hypoxic condition [5]. These results demonstrate that the *ΔmazG* mutant is unable to arrest its growth in response to growth-limiting environments.

**Figure 1.**
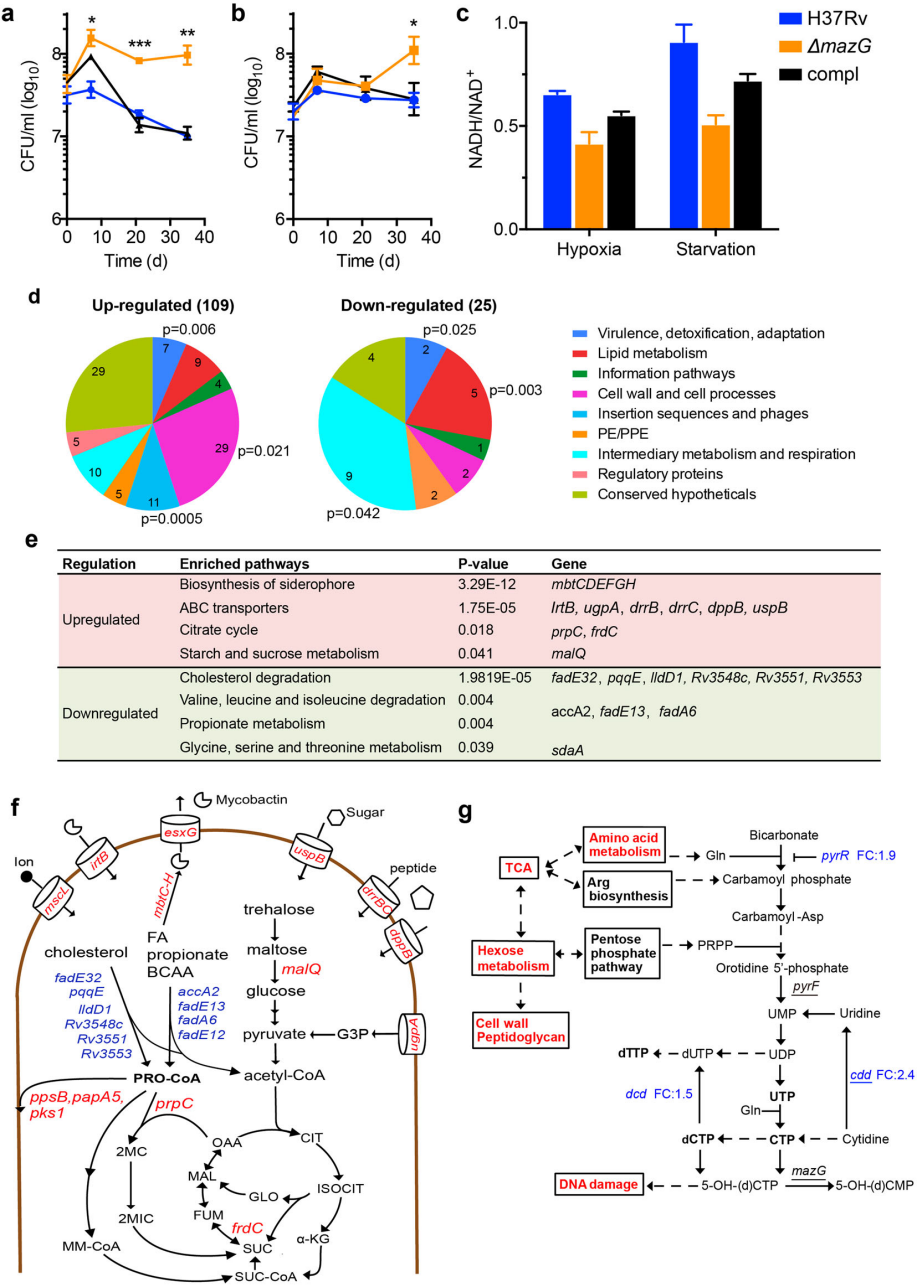
Deletion of *mazG* impairs mycobacterial metabolic adaptation in dormancy model *in vitro* and during infection of macrophages. Survival of *Mtb* stains under hypoxic (a) and nutrient-starvation (b) conditions *in vitro*. The surviving cells were quantified by counting colonies after plating. Compl, complemented mutant strain. Data shown are mean ± SE in triplicate. (c) Cellular NADH/NAD^+^ ratios in *Mtb* strains under hypoxic and nutrient-starvation conditions. Thirty-five days after hypoxic/starvation treatment intracellular NADH and NAD^+^ were extracted and measured as described in *Supplementary Materials*. Data shown are mean ± SE in duplicate. (d) Functional categories of the differentially expressed genes in the Δ*mazG* mutant (increase or decrease with fold change >2 vs wt) at 1-day post-infection of THP-1 macrophages. (e) Enrichment analyses of the differentially expressed genes in the Δ*mazG* mutant according to KEGG pathway. (f) Graphic representation of global metabolic changes in the Δ*mazG* mutant deduced from enriched pathways according to TubercuList classification and KEGG pathway. Genes showed upregulation and downregulation (Δ*mazG vs* wt, fold change >2) are indicated in red type and blue type, respectively. FA, fatty acids; 2MC, 2 methylcitrate; 2MIC, 2 methylisocitrate; MM-CoA, methylmalonyl CoA; CIT, citrate; ISOCIT, isocitrate; α-KG, alpha-ketoglutarate; SUC, succinic acid; FUM, fumarate; MAL, malic acid; OAA, oxaloacetate; GLO, glyoxylate; G3P, glycerol 3-phosphate. (g) Graphic illustration of pyrimidine metabolism and its interplay with other metabolic pathways. Metabolic pathways and genes involved in pyrimidine metabolism that showed significantly changed expression in the Δ*mazG* mutant are indicated in red type and blue type, respectively. Genes required for growth arrest of *Mtb* under hypoxic condition (Ref. [5]) are underlined. FC, fold change. *p < .05, **p < .01, ***p < .001.

Our previous studies demonstrated that the deletion of *mazG* could result in the accumulation of an oxidized pyrimidine nucleotide in mycobacteria, which may affect nucleotide homeostasis and cellular metabolism [3,4]. To investigate whether the observed growth advantage phenotype is accompanied with altered cellular metabolism, we assessed the bacterial NADH/NAD^+^ redox balance, given that the regeneration of reducing equivalents is critical for the maintenance of cellular metabolism in nongrowing *Mtb* [2]. As shown in Figure 1(c), the Δ*mazG* mutant showed decreased NADH/NAD^+^ ratios compared with that of the wild-type *Mtb*, indicating that the cellular redox balance is shifted towards an oxidizing state [2]. These results indicate that MazG-mediated maintenance of pyrimidine nucleotide pool plays a crucial role in the metabolic adaptation of nongrowing *Mtb*.

A recent study demonstrated that the stress environment of macrophages could induce nongrowing or slow-growing forms of *Mtb* [6]. To investigate whether the deletion of *mazG* affects metabolic adaptation of *Mtb* during infection, we employed RNA-seq to profile the global transcriptional response of wild-type *Mtb* and the Δ*mazG* mutant at 1-day post-infection of human macrophage-like THP-1 cells (Supplementary Figure S1), which was when the Δ*mazG* mutant begins to show reduced survival compared with that of wild type [3]. Totally, 109 upregulated and 25 downregulated genes with fold change >2 were identified in the Δ*mazG* mutant compared with wild-type *Mtb* (Dataset S1). For a global view of key regulational changes, differentially regulated genes were grouped into functional categories according to TubercuList server classification (Figure 1(d)). Among the upregulated genes, the most significantly enriched categories are insertion sequences and phages (*p* = .0005). This transcriptional signature is an indication of DNA instability in the Δ*mazG* mutant, given that the induction of transposase was shown to be a common response to DNA damage in *Mtb* and *Escherichia coli* [7,8]. Moreover, the upregulation of genes involved in DNA maintenance (*cas1*/*2*, *mrr*, *ung*, *ligA* and *mutY*) also reflects increased DNA damage in the Δ*mazG* mutant (Dataset S1). Significantly upregulated category was also observed for detoxification and adaptation processes, such as *mazEF*, *vapC* and *ephE* (involves in detoxification of oxidatively damaged lipids). Together, these transcriptional signatures corroborate the genome safeguarding and antioxidative roles of MazG [3,4].

Among the differentially regulated genes, functional categories relative to metabolism were also significantly enriched, including cell wall and cell processes (*p* = .021), intermediary metabolism and respiration (*p* = .042) and lipid metabolism (*p* = .003) (Figure 1(d)). To further delineate the metabolic changes, we performed pathway enrichment analyses according to the KEGG pathway with manual inspection. Significantly upregulated metabolic pathways include biosynthesis of mycobactin (a molecule that scavenges iron from the environment), ABC transporter, citrate cycle and starch and sucrose metabolism, whereas degradation of cholesterol [9] and branch chain amino acids (BCAAs), propionate metabolism and glycine, serine and threonine metabolism were significantly downregulated (Figure 1(e)). Overall, these results indicate that the deletion of *mazG* impacts cellular metabolism of *Mtb* during infection. The mechanism that underlies the observed metabolic changes due to *mazG* depletion remains to be determined. However, the upregulation of *cdd* (cytidine deaminase), *dcd* (dCTP deaminase) and *pyrR* (repressor of pyrimidine synthesis) in the Δ*mazG* mutant [10], taken together with the characterized role of MazG in eliminating oxidized dCTP, suggests that the observed metabolic changes may stem from dysfunction of pyrimidine metabolism.

The most intriguing metabolic change in the Δ*mazG* mutant is the simultaneous downregulation of catabolic pathways that could give rise to propionyl-CoA, a toxic metabolic intermediate generated during degradation of cholesterol, BCAA and odd chain fatty acids (Figure 1(e–f)). Although mycobacterial persistence requires the utilization of host cholesterol, *Mtb* is extraordinarily sensitive to increases in the propionyl-CoA pool unless further detoxified [9,11]. Intriguingly, genes that belong to the propionyl-CoA detoxification pathways, including the methylcitrate cycle (*prpC* and *prpR*) and synthesis of methyl-branched lipids (*ppsB*, *papA5*, *pks1* and *drrBC*), were upregulated in the Δ*mazG* mutant (Figure 1(f)). Thereafter, these transcriptional changes reflect a unique metabolic state that could alleviate the metabolic poisoning by propionyl-CoA. In addition, accompanying with the downregulation of cholesterol and BCAA catabolic pathways, genes involved in the uptake of nutrients other than lipids, including sugar (*uspB*), glycerol-3-phosphate (*ugpA*) and peptides (*drrBC* and *dppB*), as well as catabolism of disaccharide (*malQ*) were significantly upregulated in the Δ*mazG* mutant (Figure 1(e–f)). Together, these transcriptional signatures reflect a switch in the usage of carbon sources in the Δ*mazG* mutant during infection [1].

Compared with wild-type *Mtb*, several genes involved in cell cycle control were upregulated in the Δ*mazG* mutant. For instance, the expression of the resuscitation-promoting factor (*rpfE*), which is implicated in the resuscitation of mycobacteria from dormancy via a mechanism of cell wall remodeling [12], was increased 2.4-fold. Moreover, the upregulation of *malQ* reflects an increased usage of maltose derived from trehalose (Figure 1(f)), which was shown to be a critical carbon source required for the re-initiation of *de novo* synthesis of peptidoglycan for cell cycle re-entry in hypoxic nongrowing *Mtb* [13]. In line with this, we found that *murB*, the gene involved in synthesis UDP-N-acetylmuramic acid (a precursor of peptidoglycan), was also upregulated (1.7-fold) in the Δ*mazG* mutant (Dataset S1). Expression of *whiB5*, which encodes a transcriptional regulator involved in the resume of growth after reactivation from chronic infection and maintenance of metabolic activity following prolonged starvation [14], was increased in the Δ*mazG* mutant as well. In sum, these transcriptional changes thus reflect a metabolic state observed during cell cycle re-entry [1]. In this connection, it is worth noting that genes involved in iron acquisition (*mbtCDEFGH* and *irtB*) were significantly upregulated (Figure 1(e)). In addition, the upregulation of fumarate reductase (*frdC*, 7.2-fold) in the Δ*mazG* mutant may result in increased anaerobic respiration via fermentation of fumarate to succinate (Figure 1(f)), a biochemical process that was shown to be essential for viability of nongrowing *Mtb* by sustain membrane potential, ATP synthesis and providing of biosynthetic precursors [15]. In line with this observation, we found that under hypoxic condition both the isocitrate lyase and the *frdC* showed increased expression in the Δ*mazG* mutant compared with that of wild-type *Mtb* (Supplementary Figure S2).

Increasing evidence is revealing the strong impact of bacterial metabolism on the mycobacterial life cycle, pathogenicity and immunoreactivity in the host [1]. However, as an important aspect of cellular metabolism, the mechanism of nucleotide metabolism and its interplay with other metabolic pathways in nongrowing *Mtb* remains unclear (Figure 1(g)). Our study reveals that the housecleaning of pyrimidine nucleotide pool plays a unique role in mycobacterial metabolic adaptation and growth control under growth-limiting environments. These results, taken together with the previous transposon library-screening study showing that the inactivation of *Mtb pyrF* and *cdd* (all are involved in pyrimidine metabolism) resulted in failure to arrest growth under hypoxic dormancy model [5], provide evidence that pyrimidine metabolism is a metabolic checkpoint during mycobacterial adaptation to nongrowing state. Further studies were carried out in our laboratory to address the biochemistry and metabolic flux underlying the maintenance of pyrimidine nucleotide pool in *Mtb* and evaluate whether pyrimidine metabolism serves as a potential target for intervention of bacterial persistence.

Supplementary Information accompanies the manuscript on the Emerging Microbes & Infections website http://www.nature.com/emi
